# Role of integrin β1 and tenascin C mediate TGF-SMAD2/3 signaling in chondrogenic differentiation of BMSCs induced by type I collagen hydrogel

**DOI:** 10.1093/rb/rbae017

**Published:** 2024-02-24

**Authors:** Yuanjun Huang, Miao Sun, Zhenhui Lu, Qiuling Zhong, Manli Tan, Qingjun Wei, Li Zheng

**Affiliations:** Guangxi Engineering Center in Biomedical Materials for Tissue and Organ Regeneration, The First Affiliated Hospital of Guangxi Medical University, Guangxi Medical University, Nanning 530021, China; Collaborative Innovation Centre of Regenerative Medicine and Medical BioResource Development and Application Co-Constructed by the Province and Ministry, First Affiliated Hospital of Guangxi Medical University, Guangxi Medical University, Nanning 530021, China; Department of Trauma Orthopedic and Hand Surgery, The First Affiliated Hospital of Guangxi Medical University, Nanning 530021, China; Guangxi Engineering Center in Biomedical Materials for Tissue and Organ Regeneration, The First Affiliated Hospital of Guangxi Medical University, Guangxi Medical University, Nanning 530021, China; Collaborative Innovation Centre of Regenerative Medicine and Medical BioResource Development and Application Co-Constructed by the Province and Ministry, First Affiliated Hospital of Guangxi Medical University, Guangxi Medical University, Nanning 530021, China; Guangxi Engineering Center in Biomedical Materials for Tissue and Organ Regeneration, The First Affiliated Hospital of Guangxi Medical University, Guangxi Medical University, Nanning 530021, China; Collaborative Innovation Centre of Regenerative Medicine and Medical BioResource Development and Application Co-Constructed by the Province and Ministry, First Affiliated Hospital of Guangxi Medical University, Guangxi Medical University, Nanning 530021, China; Guangxi Key Laboratory of Regenerative Medicine, The First Affiliated Hospital of Guangxi Medical University, Guangxi Medical University, Nanning 530021, China; Life Science Institute, Guangxi Medical University, Nanning 530021, China; Guangxi Engineering Center in Biomedical Materials for Tissue and Organ Regeneration, The First Affiliated Hospital of Guangxi Medical University, Guangxi Medical University, Nanning 530021, China; Collaborative Innovation Centre of Regenerative Medicine and Medical BioResource Development and Application Co-Constructed by the Province and Ministry, First Affiliated Hospital of Guangxi Medical University, Guangxi Medical University, Nanning 530021, China; Guangxi Engineering Center in Biomedical Materials for Tissue and Organ Regeneration, The First Affiliated Hospital of Guangxi Medical University, Guangxi Medical University, Nanning 530021, China; Collaborative Innovation Centre of Regenerative Medicine and Medical BioResource Development and Application Co-Constructed by the Province and Ministry, First Affiliated Hospital of Guangxi Medical University, Guangxi Medical University, Nanning 530021, China; Guangxi Key Laboratory of Regenerative Medicine, The First Affiliated Hospital of Guangxi Medical University, Guangxi Medical University, Nanning 530021, China; Life Science Institute, Guangxi Medical University, Nanning 530021, China; Department of Trauma Orthopedic and Hand Surgery, The First Affiliated Hospital of Guangxi Medical University, Nanning 530021, China; Guangxi Engineering Center in Biomedical Materials for Tissue and Organ Regeneration, The First Affiliated Hospital of Guangxi Medical University, Guangxi Medical University, Nanning 530021, China; Collaborative Innovation Centre of Regenerative Medicine and Medical BioResource Development and Application Co-Constructed by the Province and Ministry, First Affiliated Hospital of Guangxi Medical University, Guangxi Medical University, Nanning 530021, China; Guangxi Key Laboratory of Regenerative Medicine, The First Affiliated Hospital of Guangxi Medical University, Guangxi Medical University, Nanning 530021, China; Life Science Institute, Guangxi Medical University, Nanning 530021, China

**Keywords:** collagen hydrogel, chondrogenic induction, integrin β1, tenascin C, TGF-SMAD2/3 signaling

## Abstract

Cartilage defects may lead to severe degenerative joint diseases. Tissue engineering based on type I collagen hydrogel that has chondrogenic potential is ideal for cartilage repair. However, the underlying mechanisms of chondrogenic differentiation driven by type I collagen hydrogel have not been fully clarified. Herein, we explored potential collagen receptors and chondrogenic signaling pathways through bioinformatical analysis to investigate the mechanism of collagen-induced chondrogenesis. Results showed that the super enhancer-related genes induced by collagen hydrogel were significantly enriched in the TGF-β signaling pathway, and integrin-β1 (ITGB1), a receptor of collagen, was highly expressed in bone marrow mesenchymal stem cells (BMSCs). Further analysis showed genes such as COL2A1 and Tenascin C (TNC) that interacted with ITGB1 were significantly enriched in extracellular matrix (ECM) structural constituents in the chondrogenic induction group. Knockdown of ITGB1 led to the downregulation of cartilage-specific genes (SOX9, ACAN, COL2A1), SMAD2 and TNC, as well as the downregulation of phosphorylation of SMAD2/3. Knockdown of TNC also resulted in the decrease of cartilage markers, ITGB1 and the SMAD2/3 phosphorylation but overexpression of TNC showed the opposite trend. Finally, *in vitro* and *in vivo* experiments confirmed the involvement of ITGB1 and TNC in collagen-mediated chondrogenic differentiation and cartilage regeneration. In summary, we demonstrated that ITGB1 was a crucial receptor for chondrogenic differentiation of BMSCs induced by collagen hydrogel. It can activate TGF-SMAD2/3 signaling, followed by impacting TNC expression, which in turn promotes the interaction of ITGB1 and TGF-SMAD2/3 signaling to enhance chondrogenesis. These may provide concernful support for cartilage tissue engineering and biomaterials development.

## Graphical Abstract

**Figure:**
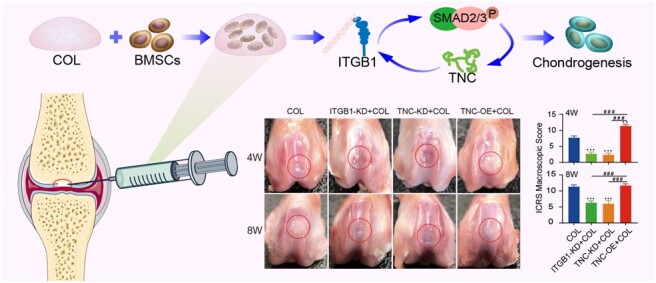

## Introduction

Cartilage defects always progress to osteoarthritis, which may greatly reduce the quality of life of affected patients [1]. The most common surgical treatment for cartilage repair includes microfracture treatment to activate the migration of mesenchymal stem cells to the defect site [2], but the regenerated tissue is mainly fibrocartilage, unmatched with hyaline cartilage in joints [3]. Stem cell-based therapy by using mesenchymal stem cells (MSCs) holds promise in the therapy of cartilage defects due to their multipotency and immunomodulatory effects that can alleviate inflammation. However, the clinical application of MSC therapy for cartilage repair has encountered difficulties due to challenges in large-scale production, maintaining viability in pathological tissue sites and limited therapeutic biological activity [4]. Fortunately, tissue engineering based on green nanomaterials [5, 6] and bioactive materials can address the aforementioned issues [4].

Characterized by good biocompatibility, degradability, low immunogenicity and cartilage-mimicking properties, collagen type I has been widely used in cartilage repair. It has been reported that collagen hydrogel has inherent inductivity and may provide a suitable environment and aggregate the signal molecule for the chondrogenic differentiation of BMSCs without exogenous growth factors both *in vitro* and *in vivo* [7, 8]. From the perspective of material properties, the chondrogenic potential of type I collagen hydrogel depends on multiple properties, like viscoelasticity, which plays an important regulatory factor of cell-matrix interactions [9], mechanical strength, which can affect the proliferation space of cells [10], the fiber structure, which influences chondrogenic differentiation by regulating factors such as mass transfer, protein adsorption, degradability and contraction [11, 12], the surface charge of hydrogels, which affects their hydrophilicity, protein diffusion and binding, which consequently impacts the adhesion and spreading of BMSCs on their surface [13] and there are reported that type I collagen hydrogels can support the microenvironment with cell–ECM interaction and migration space mediated by N-cadherin, beneficial for chondrogenesis [14–16]. Xiao *et al.* reported that type I collagen hydrogel with faster-relaxing viscoelasticity promoted cell–matrix interactions and eventually facilitated long-term chondrogenesis, similar to ROCK inhibitors, which can mitigate myosin hyperactivation and cell apoptosis [9]. However, the underlying mechanism of chondrogenic induction by type I collagen hydrogel involved in cell–ECM interactions has not been clarified until now.

Cells can recognize and adhere to immobilized ECM components by specific receptor–ligand types of interaction to influence subsequent cellular behavior, such as cell survival and differentiation [17, 18]. The best-known receptor of collagen is integrin, which plays a major role in mediating interactions between cells and the ECM [19]. There are at least 18 α subunits and 8 β subunits in humans, which together generate 24 integrin proteins. Among them, integrin β1 (ITGB1) is the hub subunit, which can combine with other 12 α subunits like α2β1 and α11β1 integrin [20], and it belongs to the integrin family mainly associated with chondrogenesis [21, 22]. The integrin β binds to the domain of TGF-β precursor to activate TGF-β precursor to eventually release the mature TGF-β growth factor that contributes to chondrogenesis [23, 24]. Loss of ITGB1 abolishes the ability of cells to mitigate myosin activation and would lead to failure of chondrogenic differentiation [9, 25]. Silencing of ITGB1 in MSCs abolished both osteoblastic and chondrogenic differentiation in response to substrate stiffness [26]. As the downstream of integrin β [27], TGF-β/SMAD signaling is crucial for chondrogenesis [28] and maintenance of the cartilage matrix [29]. The phosphorylation of SMAD2/3 is an important event for activating the TGF-β/SMAD signaling and its nuclear localization to initiate the chondrocyte-related gene expression [30, 31]. SMAD2/3 can upregulate the protein level of SOX9, and form transcriptional complexes with SOX9 [32, 33]. SOX9 is an important transcription factor that binds to the enhancer region of COL2A1 and is required for cartilage formation [34]. Interestingly, type I collagen is the main ECM complex in the mesenchymal condensation process that initiates cartilage formation [35, 36]. Gigout *et al.* [37] attributed cell aggregation to type I collagen and integrin β1-dependent binding, and type I collagen hydrogel can upregulate the expression level of SOX9 [13, 38–40] and increase the nuclear localization of SMAD2/3 [41]. In addition, Tenascin C (TNC) can induce phosphorylation of SMAD2 and SMAD3 [42]. Thus, ITGB1 with the regulation of TGF-β/SMAD signaling and TNC may be important in the chondrogenic differentiation driven by type I collagen hydrogel, although it has not been reported yet.

This study focused on the underlying mechanism of chondrogenesis triggered by collagen hydrogel by predicting collagen receptor pathways based on bioinformatical methods. We hypothesized that collagen hydrogel may regulate the TGF-β/SMAD signaling and TNC expression through activating ITGB1 as the collagen receptor, ultimately initiating chondrogenic differentiation. This study may establish the relationship between bioactive materials and molecular mechanisms for materials-optimized design guiding in cartilage tissue engineering.

## Materials and methods

### Public database data analysis

The gene expression profile (GSE40175) of chondrogenic inducement of bone marrow mesenchymal stem cells (BMSCs) was downloaded from the GEO database (https://www.ncbi.nlm.nih.gov/geo/query/acc.cgi?acc=GSE40175) [43]. This dataset included both the BMSCs group and the chondrocyte-induction group. Membrane-related proteins (species: Homo sapiens) were downloaded from the Membranome database (https://membranome.org/proteins, 2021 version). Expression levels of membrane proteins were analyzed in GSE40175 to obtain the top 10 membrane proteins with the highest expression levels in BMSCs by sorting from highest to lowest. Genes related to the TGF-β signaling pathway were obtained by inputting ‘TGF-β signaling pathway’ into GeneCards (https://www.genecards.org/), and non-Protein Coding genes were excluded. These genes were then subjected to protein–protein interaction analysis using the CytoscapeAPP.

### Cell extraction culture

Neonatal rabbits and SD rats used in this study were obtained from the Experimental Animal Center of Guangxi Medical University (Nanning, China). Animal surgeries were approved by the Ethics Committee of Guangxi Medical University (no: 201905900). BMSCs were extracted from the bone marrow of rabbits or Sprague Dawley (SD) rats. In brief, the limbs of the animal were obtained by sacrifice using an overdose pentobarbital sodium (Solarbio, Beijing, China). Soft tissues attached to the bones were removed. The epiphyses were cut off, and the bone marrow cavity was repeatedly flushed to obtain mononuclear cells. These cells were then seeded in plates and cultured with Dulbecco’s modified eagle medium (DMEM)/Basic (Hyclone, USA) supplemented with 10% fetal bovine serum (FBS; TIANHANG, Hangzhou, China), 100 units/ml penicillin and 100 μg/ml streptomycin (Solarbio, Beijing, China). The cells were cultured in a humidified atmosphere containing 5% CO_2_ at 37°C. After 7 days of culture, BMSCs were collected and subcultured at a density of 4 × 10^3^ cells/cm^2^ in plastic dishes. The medium was refreshed every 3 days. When the cells reached 100% of confluency, they were digested using 0.25% trypsin (Solarbio, Beijing, China) containing 0.02% EDTA. Further experiments were conducted using BMSCs at the third generation.

### ChIP-sequencing analysis

After co-culturing rabbit BMSCs with collagen hydrogel for 7 days, chromatin immunoprecipitation sequencing (ChIP-seq) technology was used to measure the signal intensity of histone H3K27ac, H3K4me3 and transcription factor CTCF [44, 45]. Super-enhancers (SEs) were identified using ROSE (rank ordering of super-enhancers), a tool that distinguishes SEs from typical enhancers by searching for genome regions highly enriched in ChIP-Seq signals. Stitching enhancers were detected by merging enhancers within 12.5 kb, and then the specific threshold of SEs was determined based on the ranking of stitching enhancer signals [45]. The genes that were closest to SEs were used for KEGG pathway analysis based on the KEGG database (Kyoto Encyclopedia of Genes and Genomes, http://www.genome.jp/kegg). The significance of pathways was represented by *P* values, where smaller *P* values indicate more significance (*P* values cutoff was set to 0.05). Sequencing and super-enhancer analysis services were provided by Aksomics (website: www.aksomics.com).

### Cell transfection and purification

TNC-overexpressing lentiviruses were designed and synthesized by GeneCopoeia (Guangzhou, China), and TNC and ITGB1 knockdown lentiviruses were designed and synthesized by Genechem (Shanghai, China). Lentiviruses were used for transfection according to the manufacturer’s instructions. BMSCs at first passage were collected and ITGB1 and TNC were knocked down or overexpressed by lentiviral transfection. Cells were selected and purified with puromycin after transfection for 72 h.

### Quantitative real-time polymerase chain reaction) analysis

A Hipure Total RNA Mini Kit (Magen, Guangzhou, China) was used for mRNA extraction. Primer sequences applied to quantitative real-time polymerase chain reaction (qRT-PCR) are summarized in Table 1. Standard qRT-PCR experiments were performed according to our previous study [46]. Glyceraldehyde-3-phosphate dehydrogenase (GAPDH) was used as an internal reference.

**Table 1.** Sequences of primers used in qRT-PCR

Gene	Forward primer	Reverse primer
SOX9	5′-TCCAGCAAGAACAAGCCACA-3′	5′-CGAAGGGTCTCTTCTCGCTC-3′
COL2A1	5′-GACTGTGCCTCGGAAGAACT-3′	5′-TCTGGACGTTAGCGGTGTTG -3′
ACAN	5′-GAATGGGAGCCAGCCTACAC-3′	5′-GAGAGGCAGAGGGACTTTCG-3′
TNC	5′-GTTTCCGGATTACTTATGTGCC-3′	5′-CGGATCACTTTCTTCAAATCCC-3′
ITGB1	5′-ACATTGATGACTGCTGGTTCTA-3′	5′-AATAAGAACAATTCCGGCAACC-3′
SMAD2	5′-AGACCTTCCATGCGTCACAG -3′	5′-TAGGCACTCGGCAAACACTT -3′
GAPDH	5′-TCCAGTATGACTCTACCCACG-3′	5′-CACGACATACTCAGCACCAG-3′

### Western blot

Total protein was extracted with RIPA (Biosharp, Hefei, China) and the concentrations were quantified with a BCA kit (Beyotime Biotechnology, Shanghai, China). Proteins were separated by 10% SDS page and then transferred to PVDF (Biosharp, Hefei, China) membranes. After blocking with 5% skim milk, membranes were washed three times for 5 min with TBST(Solarbio Science & Technology, Beijing, China) and the primary antibody against COL2A1 (1:1000; Santa Cruz Animal Health, USA) was used for incubation overnight. After being washed by TBST again, a secondary antibody was added and incubated for 1 h at 37°C. Bands were scanned by an Odyssey Infrared Imaging System (Odyssey, USA).

### Cartilage defect model construction

Forty male SD rats (6–8 weeks old) were used. After general anesthesia, the joint of the SD rat was exposed layer by layer, and a cartilage defect (2 mm in diameter, 1.5 mm in depth) was created to establish the cartilage defect model. Rats were randomly divided into four groups: (i) COL (BMSCs + collagen); (ii) ITGB1-KD + COL (ITGB1 knockdown-BMSCs + collagen); (iii) TNC-KD + COL (TNC knockdown-BMSCs + collagen); (iv) TNC-OE + COL (TNC overexpression-BMSCs + collagen). Cells encapsulated in the collagen hydrogel were injected into the defect site and jellified. Then the wound was sutured, and 20 000 U/100 g penicillin sodium (HEBEI YUANZHENG PHARMACEUTICAL, Shijiazhuang, China) was intramuscularly injected every day for 3 days.

### Gross morphology observation and assessment

After repairing for 4 and 8 weeks, rats were euthanized by injection of excessive pentobarbital sodium. The joints were collected and the defects were evaluated by three assessors blindly to the groups using the International Cartilage Repair Score (ICRS) system [47].

### Histological assessment

Specimens were fixed in 4% paraformaldehyde and decalcified by EDTA. After gradient dehydration, paraffin embedding, sectioning and dewaxing, sections were dyed with hematoxylin and eosin (H&E) or safranin O/fast green for histological evaluation. Images were collected by using a microscope (ECHO, USA). Histological scores reported previously were used for the cartilage repair scoring system [48].

### Immunohistochemical and immunofluorescence staining

Samples were incubated with 3% H_2_O_2_ for 10 min at 25°C to block endogenous peroxidase activity. After blocking with normal goat serum, primary antibodies against TNC (1:100; Proteintech, Wuhan, China), ITGB1 (1:100; Proteintech, Wuhan, China), COL2A1 (1:100; Proteintech, Wuhan, China), COL1A1 (1:100; Proteintech, Wuhan, China), p-SMAD2/3 (1:100; Cell Signaling, USA) and SMAD2/3 (1:100; Cell Signaling, USA) were added and incubated overnight at 4°C. For the immunohistochemical staining, a secondary antibody was incubated for 1 h, and then used DAB kit (ZSGB-BIO, Beijing, China) for development. After being stained with hematoxylin, the sections were sealed and photographed by a microscope. For the immunofluorescence staining, fluorescent dye-conjugated secondary antibodies FITC and CY3 (1:100; Bioss, Beijing, China) and 4′, 6-diamidino-2-phenylindole (DAPI; Beyotime Biotechnology, Shanghai, China) were used for binding and nuclear staining. Images were collected with a fluorescence microscope (ECHO, USA) and fluorescence intensity was semi-quantitatively analyzed by Image J.

### Statistical analysis

All tests were performed at least triplicate. Statistical analysis of all data was analyzed by SPSS 24.0 (SPSS Inc., USA). Statistical significance for the significance of differences between two groups was verified using Student’s *t*-test and multiple comparisons were determined using one-way analysis of variance (ANOVA). *P* < 0.05 was considered statistically significant.

## Results and discussion

### ITGB1 was a potential receptor for chondrogenic differentiation induced by collagen hydrogel

**Figure 1.**
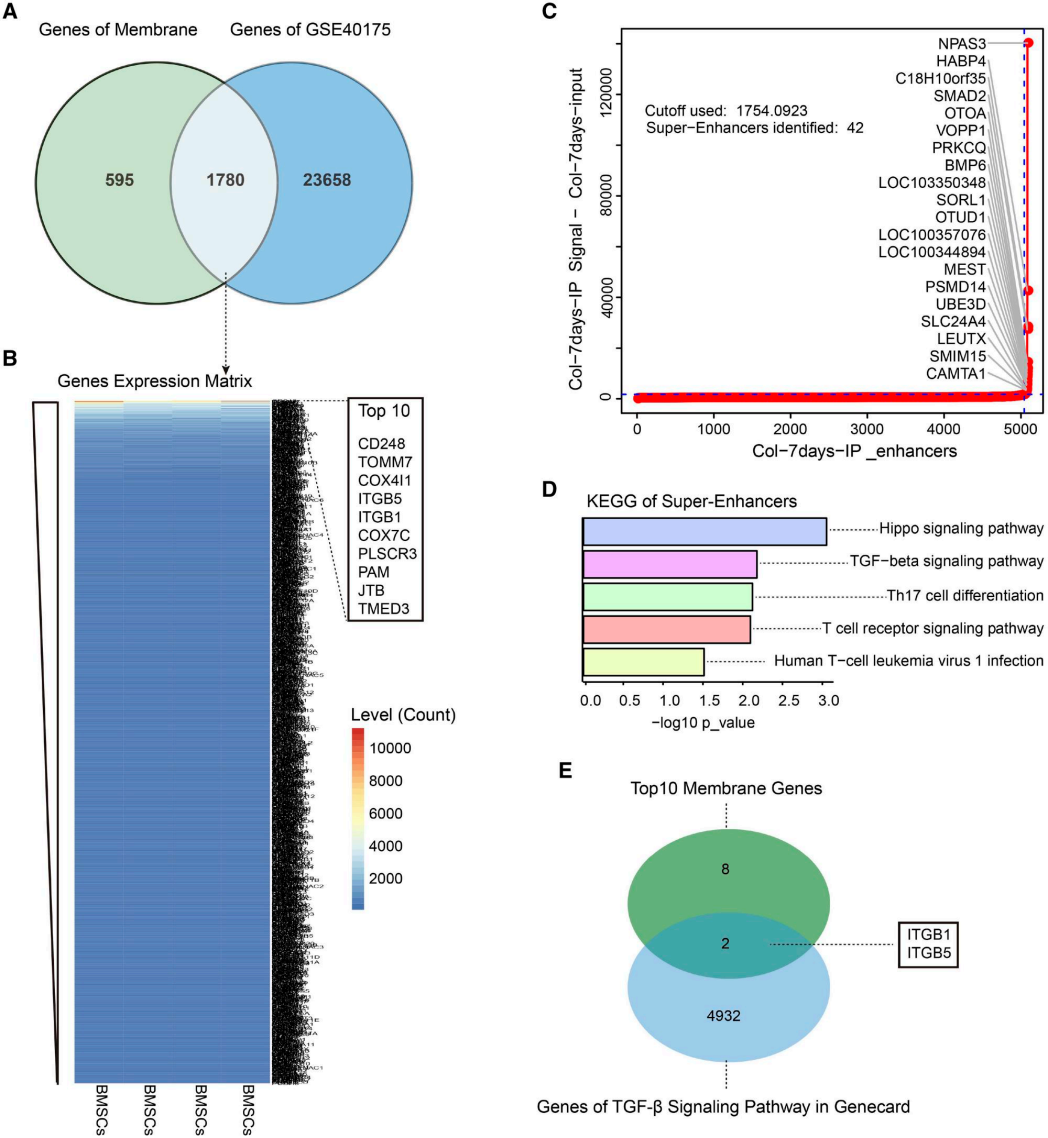
(**A**) Intersections of membrane protein-related genes and genes from GSE40175. (**B**) Expression levels of overlapped membrane protein genes and the top 10 with the highest expression levels. (**C**) The top 20 SEs in the BMSCs induced by collagen hydrogel for 7 days. (**D**) KEGG-pathway enrichment analysis of the SEs-related genes. (**E**) Intersections of the top 10 membrane protein genes and genes related to the TGF-β signaling pathway.

The intersection between Membranome database and the chondrogenic induction database GSE40175 was used to screen the potential membrane protein genes in the process of chondrogenic differentiation. A total of 1780 overlapped genes were identified (Figure 1A). The top 10 overlapped membrane protein genes including CD248, TOMM7, COX4I1, ITGB5, ITGB1, COX7C, PLSCR3, PAM, JTB and TEMD3 were identified by sorting the expression level (Figure 1B). The SEs in chondrogenic differentiation of BMSCs induced by collagen hydrogel were analyzed to screen the underlying signaling pathways. It demonstrated that a total of 42 SEs were identified in BMSCs induced by collagen hydrogel for 7 days (Supplementary file 1), and the top 20 SEs including NPAS3, HABP4, C18H10orf35, SMAD2 and so on (Figure 1C). Pathway enrichment analysis of SEs-related genes exhibited significant enrichment in the Hippo and TGF-β signaling pathways (Figure 1D). SMAD2 that involved in the canonical transduce signals of TGF-β signaling pathway plays an essential role in chondrogenesis [49]. It can regulate gene expression of SOX9 to drive chondrogenesis by forming a complex with SMAD3 and subsequent phosphorylation [50]. Gene-deficient mice showed that the deficiency of SMAD2 exhibited a greater impact on chondrogenesis than the loss of SMAD3 [49].

Considering the importance of TGF-β in chondrogenic differentiation, 4934 protein-coding genes related to the TGF-β signaling pathway from the GeneCards database were used to intersect with the top 10 membrane protein genes mentioned above. Two overlapped membrane protein genes including ITGB1 and ITGB5 that are associated with the TGF-β signaling pathway were confirmed (Figure 1E). This indicated that membrane protein receptors ITGB1 and ITGB5 that existed in the TGF-β signaling pathway may be involved in the chondrogenic differentiation of BMSCs induced by collagen hydrogel. In ITGB1-deficient mice, Integrin can potentiate the TGF-β signaling pathway by directly mediating adhesion and indirectly controlling the levels of components in the TGF-β pathway, such as SMAD [51]. It is also reported that cell–ECM interaction mediated by integrin is crucial for chondrogenesis [52].

**Figure 2.**
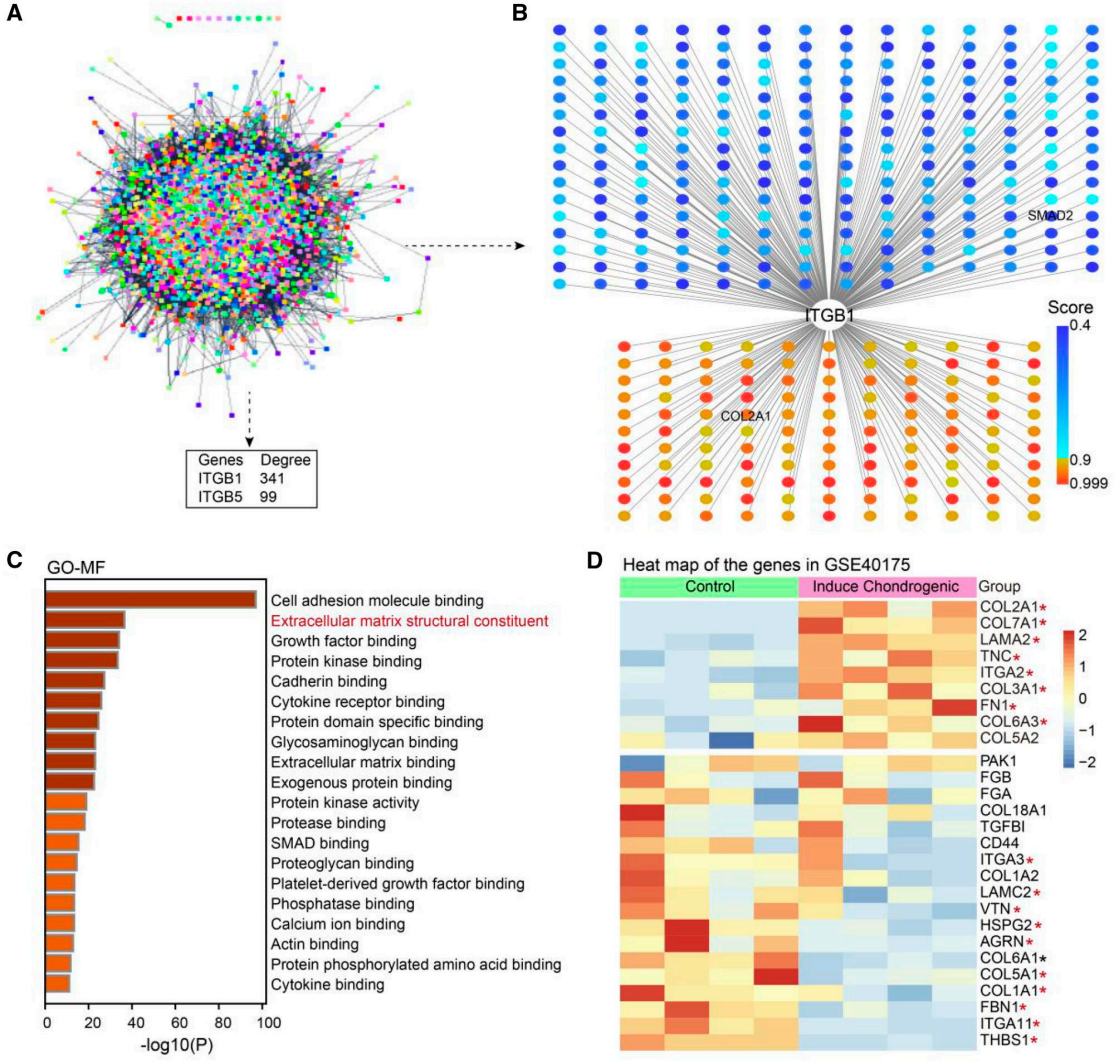
(**A**) Interaction levels of membrane protein genes (ITGB1, ITGB5) and TGF-β signaling pathway detected by protein–protein interaction analysis. (**B**) Genes in the TGF-β signaling pathway interacted with ITGB1. (**C**) Molecular functional enrichment analysis of the genes interacted with ITGB1. (**D**) Heatmap of the genes expression level in the enriched extracellular matrix structural constituents signaling pathway. *P <* *0.05 indicates that there is a significant difference in expression between the induction group and the control group.

Protein–protein interaction analysis between the 4934 protein genes in the TGF-β signaling pathway and the two membrane protein genes (ITGB1, ITGB5) was performed by CytoscapeAPP to identify the most crucial membrane protein gene related to the TGF-β signaling pathway. The interaction levels of the two membrane protein genes were calculated and ITGB1 exhibited the highest interaction degree, implying a greater contribution than ITGB5 (Figure 2A). ITGB1, not ITGB5, has been reported to mediate cell differentiation and formation of ECM [53]. And the receptors of collagen commonly contain ITGB1 instead of ITGB5 [54]. The genes that interacted with ITGB1 were extracted. As shown in Figure 2B, 341 genes including SMAD2 and COL2A1 were associated with ITGB1. These 341 genes were subjected to a molecular functional enrichment analysis to further explore molecular functions related to chondrogenic induction. Genes were mainly enriched in the GO terms of cell adhesion molecule binding and extracellular matrix structural constituents (Figure 2C). As collagen serves as an ECM-mimicking scaffold, we hypothesized that genes in the GO term of extracellular matrix structural constituents may be the key regulator signal transduction molecules. Genes in extracellular matrix structural constituents with interaction scores greater than 0.9 with ITGB1 are show in Figure 2D. Among which, COL2A1, TNC, etc. were upregulated and COL1A1, etc. were downregulated. COL2A1 is the phenotype of hyaline cartilage which mainly distributes in the joints. TNC, an ECM glycoprotein that exists in the condensing mesenchyme of embryonic cartilages, appears to play a role in early differentiation events and loss from cartilage with progressive chondrocyte maturation [55]. It can promote the chondrogenesis of MSCs *in vitro* and this effect can be inhibited by antibodies against TNC [56]. Based on these findings, we speculate that collagen hydrogel may regulate the expression of chondrogenic genes through ITGB1 mediation of TGF-β signaling, followed by initialing of TNC expression.

### ITGB1 regulated the gene expression of TNC and SMAD2 in chondrogenic differentiation induced by collagen hydrogel

**Figure 3.**
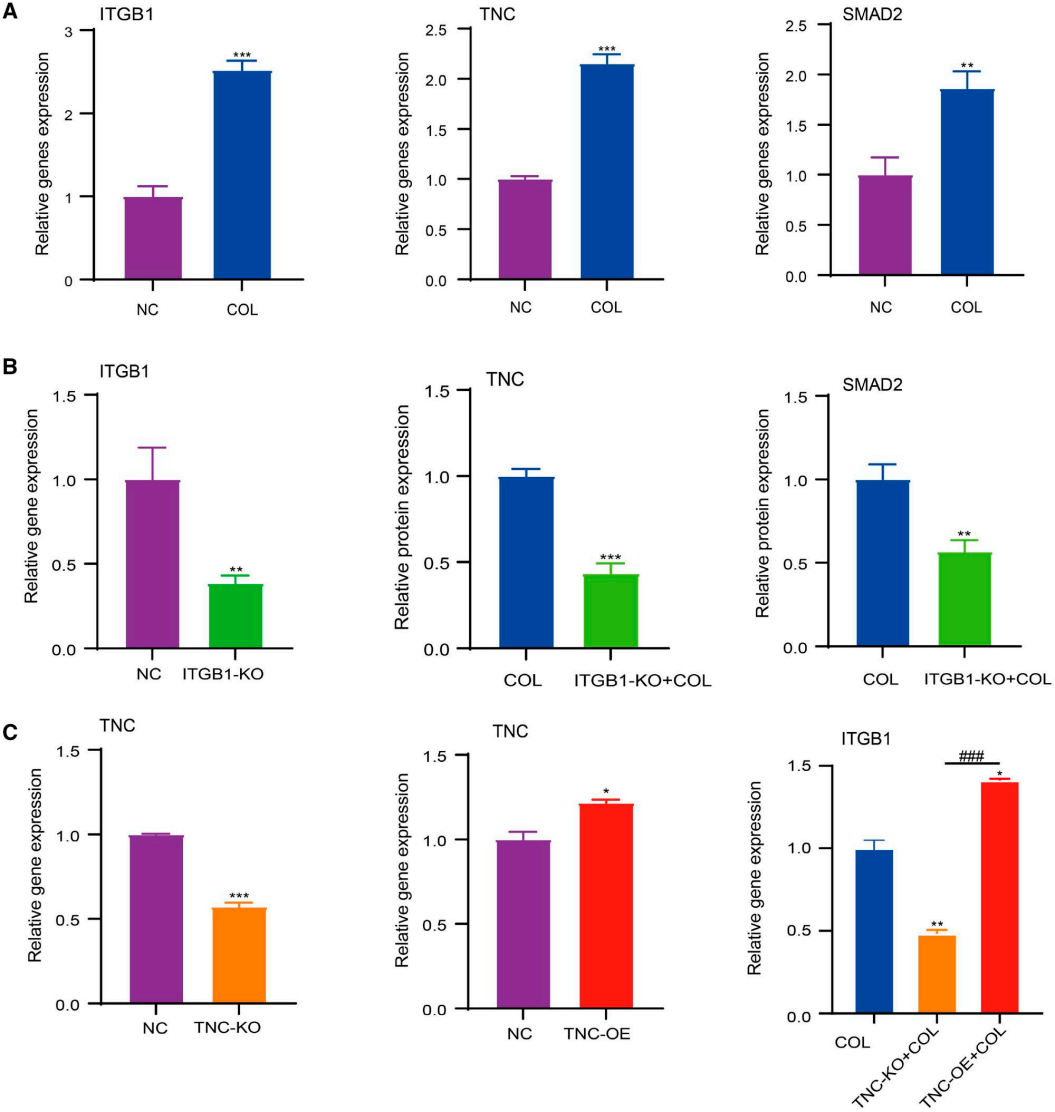
(**A**) Expression levels of ITGB1, TNC and SMAD2 in BMSCs cultured only or intra-hydrogel culture in collagen. (**B**) Expression levels of ITGB1 in normal or ITGB1 knockdown BMSCs, and the expression levels of TNC and SMAD2 in normal or ITGB1 knockdown BMSCs cultured in hydrogel collagen. (**C**) Expression level of TNC in normal, TNC knockdown or overexpression BMSCs, and the expression level of ITGB1 in normal, TNC knockdown or overexpression BMSCs cultured in collagen hydrogel (mean±SD, *n* = 3, *P ≤* *0.05, **P ≤* *0.01, ***^, ###^*P *≤* *0.001, * represents the comparison between the experimental group and the control group, and # represents the comparison between the experimental groups).

To validate the above predictive results, the gene expression levels of ITGB1, SMAD2 and TNC were detected. The levels of ITGB1, TNC and SMAD2 in BMSCs were upregulated in the induction of collagen hydrogel (Figure 3A), which was consistent with the predictive results (Figure 2). As ITGB1 is a potential receptor of collagen, it was knocked down to verify whether it can affect the expression of TNC and SMAD2 during the chondrogenesis induced by collagen hydrogel. The result showed that ITGB1 was knocked down successfully and TNC and SMAD2 were downregulated by knockdown of ITGB1 in the induction of collagen hydrogel (Figure 3B), implying that ITGB1 could regulate the gene expression of TNC and SMAD2 for chondrogenesis. It was reported that hydrogel viscosity promoted the activation and nuclear translocation of SMAD2/3 to elevate the chondrogenesis by mediating ITGB1 [57]. In cultured human dermal fibroblasts, DNA affinity precipitation assay revealed that Phospho-SMAD2/3 could bind to the promoter of TNC in a transient and specific manner, thereby regulating TNC expression [58].

The knockdown and overexpression of TNC were further performed to detect the function of TNC in the process of chondrogenesis induced by collagen hydrogel. It showed that TNC was knocked down and overexpressed successfully (Figure 3C). Intra-hydrogel culture in collagen, knockdown of TNC downregulated the expression of ITGB1, while overexpression of TNC upregulated the expression of ITGB1 (Figure 3C). It was reported that ITGB1 could also be promoted by TNC [59] to form a positive feedback loop to activate TGF-β signaling [60, 61], which is consistent with our findings. These demonstrated that cells with ITGB1 and TNC knockdown, as well as TNC overexpression were successfully constructed. ITGB1 regulated the gene expression of TNC and SMAD2, and TNC could in turn promote the expression of ITGB1.

### ITGB1 and TNC affect the TGF-SMAD2/3 signaling in chondrogenic differentiation induced by collagen hydrogel

**Figure 4.**
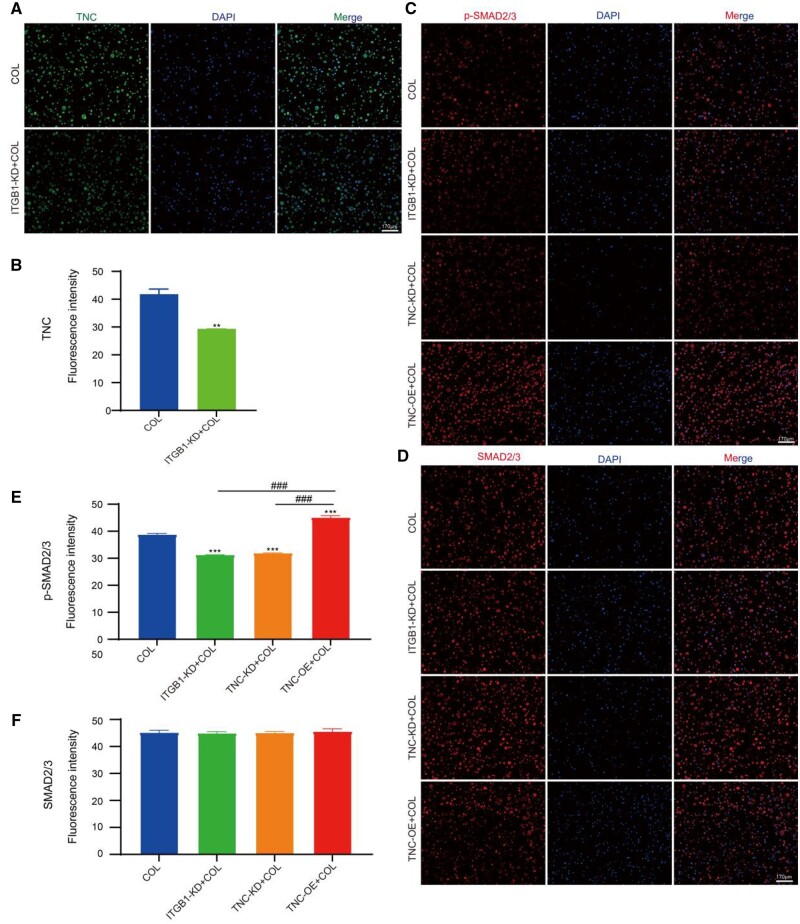
(**A**) Immunofluorescence staining for TNC in COL and ITGB1+KD+COL groups. (**B**) Semi-quantitation of fluorescence intensity in (A). (**C** and **D**) immunofluorescence staining for p-SMAD2/3 and SMAD2/3 in COL, ITGB1+KD+COL, TNC+KD+COL and TNC+OE+COL groups. (**E** and **F**) Semi-quantitation of fluorescence intensity in (C) and (D) (scale bar: 170 μm; mean ± SD, *n* = 3; **P ≤* *0.01, ***^, ###^*P *≤* *0.001, * represents the comparison between the experimental group and the control group, and # represents the comparison between the experimental groups).

To clarify the role of ITGB1 and TNC in mediating TGF-SMAD2/3 signaling in the chondrogenic differentiation induced by collagen hydrogel, the expression of TNC, SMAD2/3 and p-SMAD2/3 was detected by immunofluorescence staining. The fluorescence intensity of TNC was decreased when the knockdown of ITGB1 (Figure 4A and B), indicating it was the downstream of ITGB1. The phosphorylation level of SMAD2/3 (p-SAMD2/3) which is a key event in TGF-β signaling-mediated chondrogenesis was decreased in the knockdown of ITGB1 or TNC and promoted by overexpression of TNC (Figure 4C and E). However, no changes in the expression level of SMAD2/3 protein were observed, suggesting the ITGB1 and TNC can’t affect the protein expression level of SMAD2/3 (Figure 4D and F). Consistent with the findings of Mia, etc., ITGB1 deletion downregulated SMAD2/3 phosphorylation, but the total expression of SMAD-2/3 showed no significant change [62]. These results indicated that the expression level of TNC can be affected by ITGB1. ITGB1 and TNC influence the activation of TGF-SMAD2/3 signaling.

### ITGB1 and TNC enhanced chondrogenic differentiation of BMSCs induced by collagen hydrogel

**Figure 5.**
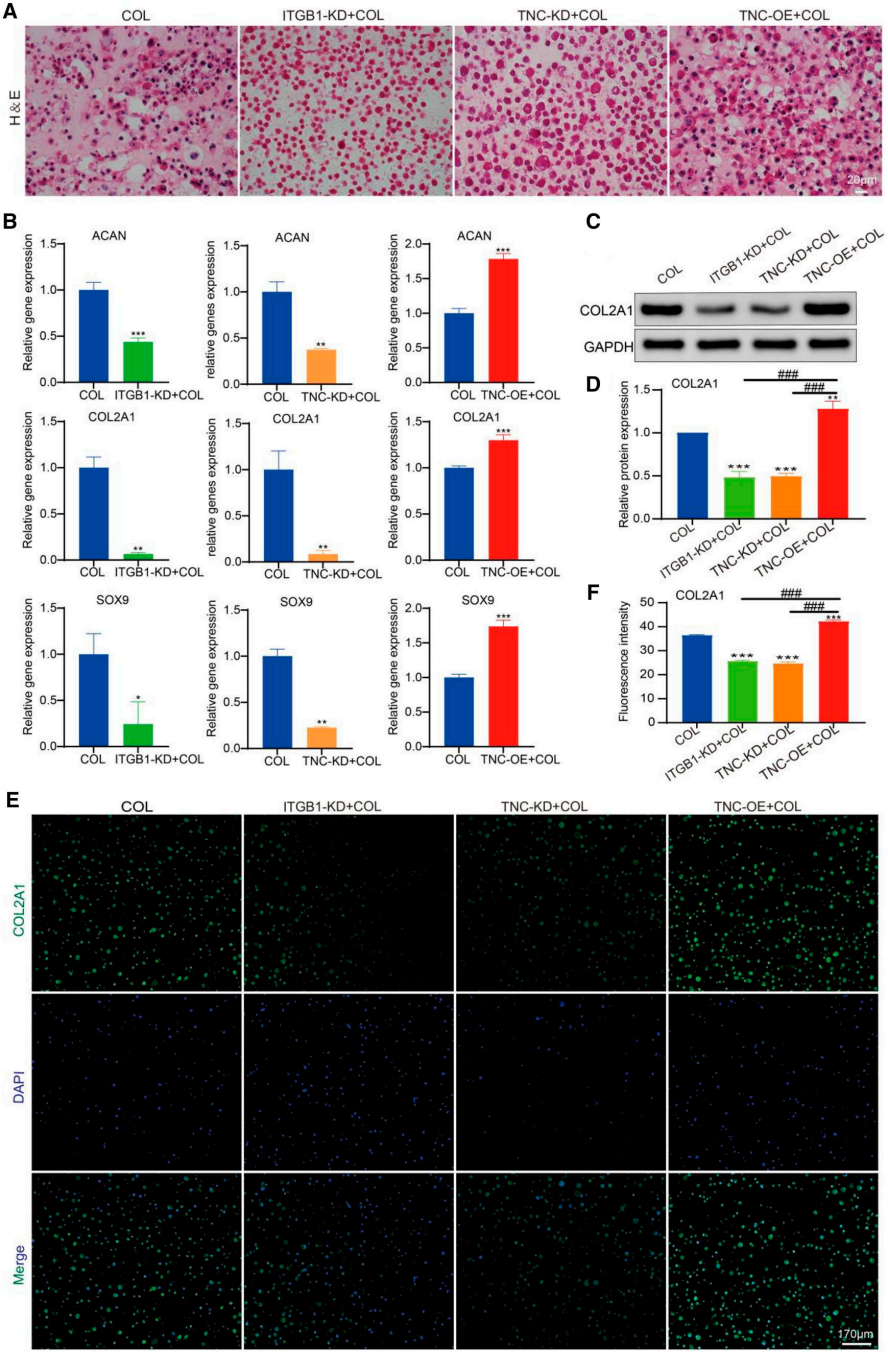
(**A**) H&E staining. (**B**) Gene expression levels of ACAN, COL2A1 and SOX9. (**C**) Western blot detect the protein expression of COL2A1. (**D**) Semi-quantitation of gray level in (C). (**E**) Immunofluorescence staining for COL2A1. (**F**) Semi-quantitation of fluorescence intensity in (E) (scale bars: 170 μm. mean± SD, *n* = 3; *P ≤* *0.05, **P ≤* *0.01, ***^, ###^*P *≤* *0.001. * represents the comparison between the experimental group and the control group, and # represents the comparison between the experimental groups).

Normal, ITGB1 knockdown, TNC knockdown or TNC overexpression BMSCs were intra-hydrogel cultured in collagen to measure the effect of ITGB1 and TNC on chondrogenic differentiation of BMSCs induced by collagen hydrogel. H&E staining showed that in the induction of collagen hydrogel, chondrocytes with a typical lacunar structure were observed (Figure 5A), indicating the chondrogenesis of BMSCs. However, cells with a typical lacunar structure were almost can’t observed in the ITGB1 or TNC knockdown groups. Overexpression of TNC recovered the chondrogenic effect of collagen hydrogel. The expression levels of chondrogenic genes (ACAN, COL2A1 and SOX9) were also enhanced by intra-hydrogel cultured in collagen. They were downregulated by knockdown of ITGB1 or TNC and rescued by overexpression of TNC (Figure 5B). Furthermore, the protein expression levels of COL2A1 which is the main component of cartilage ECM were analyzed by western blot and immunofluorescence staining. Compared to the COL group, the expression of COL2A1 protein was down-regulated in the ITGB1-KD+COL and TNC-KD+COL groups but upregulated in the TNC-OE+COL group (Figure 5C–F). And there was also direct evidence that the ITGB1 gene was positively correlated with the expression of COL2A1, ACAN and SOX9, and has a function to promote chondrogenic differentiation of MSCs [63]. These results indicated that ITGB1 and TNC were involved in the chondrogenic differentiation of BMSCs induced by collagen hydrogel.

### ITGB1 and TNC promoted cartilage regeneration induced by collagen hydrogel *in vivo*

**Figure 6.**
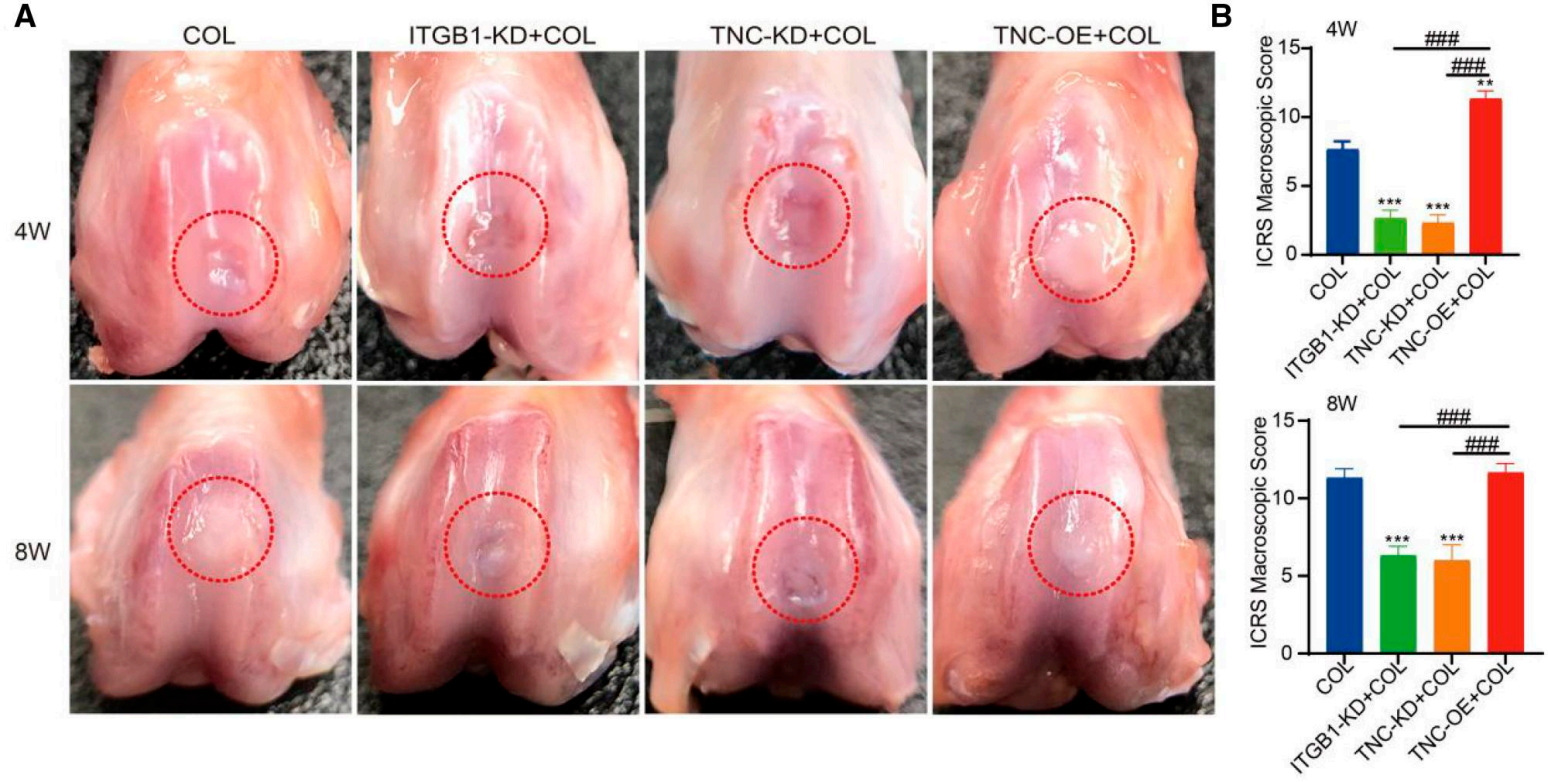
Macroscopic appearance (**A**) and (**B**) ICRS macroscopic scores of the regenerated cartilage at 4 and 8 weeks post-surgery (mean ± SD, *n* = 3; **P ≤* *0.001, ***^, ###^*P *≤* *0.001. * represents the comparison between the experimental group and the control group, and # represents the comparison between the experimental groups).

To evaluate the effect of ITGB1 and TNC in cartilage regeneration *in vivo*, a rat articular cartilage defect model was constructed. No postoperative infection was observed in rats during the therapeutic period. The representative macroscopic appearances of regenerated cartilage from four groups after 4 weeks of treatment are shown in Figure 6A. At week 4 after treatment, the defects in the COL group were almost filled with regenerated tissues. Compared to the COL group, the defects in the ITGB1-KD+COL and TNC-KD+COL groups were still obvious. In the TNC-OE+COL group, the defects were fulfilled with regenerative tissue although a clear boundary was still clear between new and orthotopic tissue. After 8 weeks of treatment, the regenerative tissues in the COL group completely repaired the defect with a smooth appearance and were well intergraded with the normal tissue. Defects repair in ITGB1-KD+COL and TNC-KD+COL groups were still insufficient, as evidenced by regenerative tissues haven’t completely filled. Regenerative tissue in the TNC-OE+COL group exhibited well integrated with the surrounding tissues with smooth surface and closed to the normal cartilage. It was reported that the deficient of ITGB1 could lead to the development of chondrodysplasia [64], and knocking down TNC leads to delayed repair of cartilage defects [65] while supplementing TNC promotes the repair of cartilage defects [66, 67]. The ICRS macroscopic scores were performed to further evaluate regenerated cartilage. The highest score was shown in the TNC-OE+COL group (10.6 ± 0.6), followed by the COL group (8.0 ± 1.0) after implantation for 4 weeks (Figure 6B). The difference between ITGB1-KD+COL (4.6 ± 0.6) and TNC-KD+COL (3.6 ± 0.6) groups was not significant. After 8 weeks of implantation, the ICRS macroscopic score in the TNC-OE+COL group (12.0 ± 0.0) was much the same as the COL group (11.6 ± 0.6), which was much higher than the other two groups.

**Figure 7.**
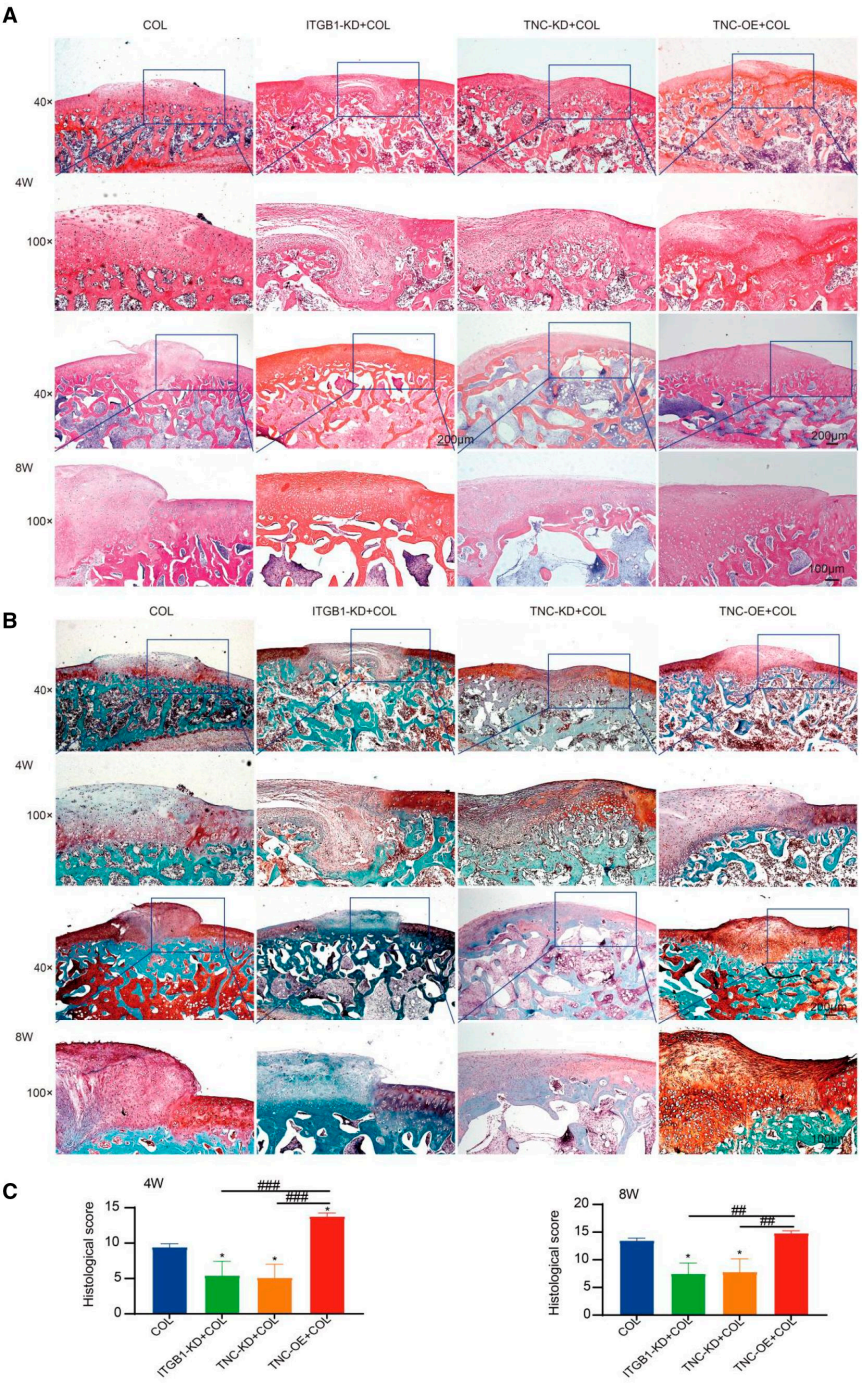
H&E staining (**A**), safranine O staining (**B**) and histological score (**C**) of repaired cartilage at 4 and 8 weeks post-surgery (scale bars: 200 and 100 μm; mean ±SD, *n* = 3; *P ≤* *0.05, ^##^*P *≤* *0.01, ^###^*P *≤* *0.001. * represents the comparison between the experimental group and the control group, and ^#^ represents the comparison between the experimental groups).

Histological analysis stained for H&E and safranine O was carried out to investigate the effect of ITGB1 and TNC in cartilage regeneration mediated by collagen hydrogel. As exhibited in Figure 7A and B, the regenerated tissues in the COL and TNC-OE+COL groups had typical hyaline-like characteristics with uniformly distributed cells of lacunar structure and GAG deposition after 4 and 8 weeks of treatment. In contrast, the regenerated tissues in ITGB1-KD+COL and TNC-KD+COL groups were fibrous with less GAG generation. Furthermore, the histological scores according to the H&E and safranine O staining were decreased in ITGB1-KD+COL (5.3 ± 2.1 and 7.3 ± 2.1 in 4 and 8 weeks) and TNC-KD+COL (5 ± 2 and 7.6 ± 2.5 in 4 and 8 weeks) groups compared to COL group (Figure 7C). Over-expression of TNC reversed the cartilage regeneration as indicated by the histological scores were increased to 13.6 ± 0.6 and 14.6 ± 0.6 in 4 and 8 weeks.

**Figure 8.**
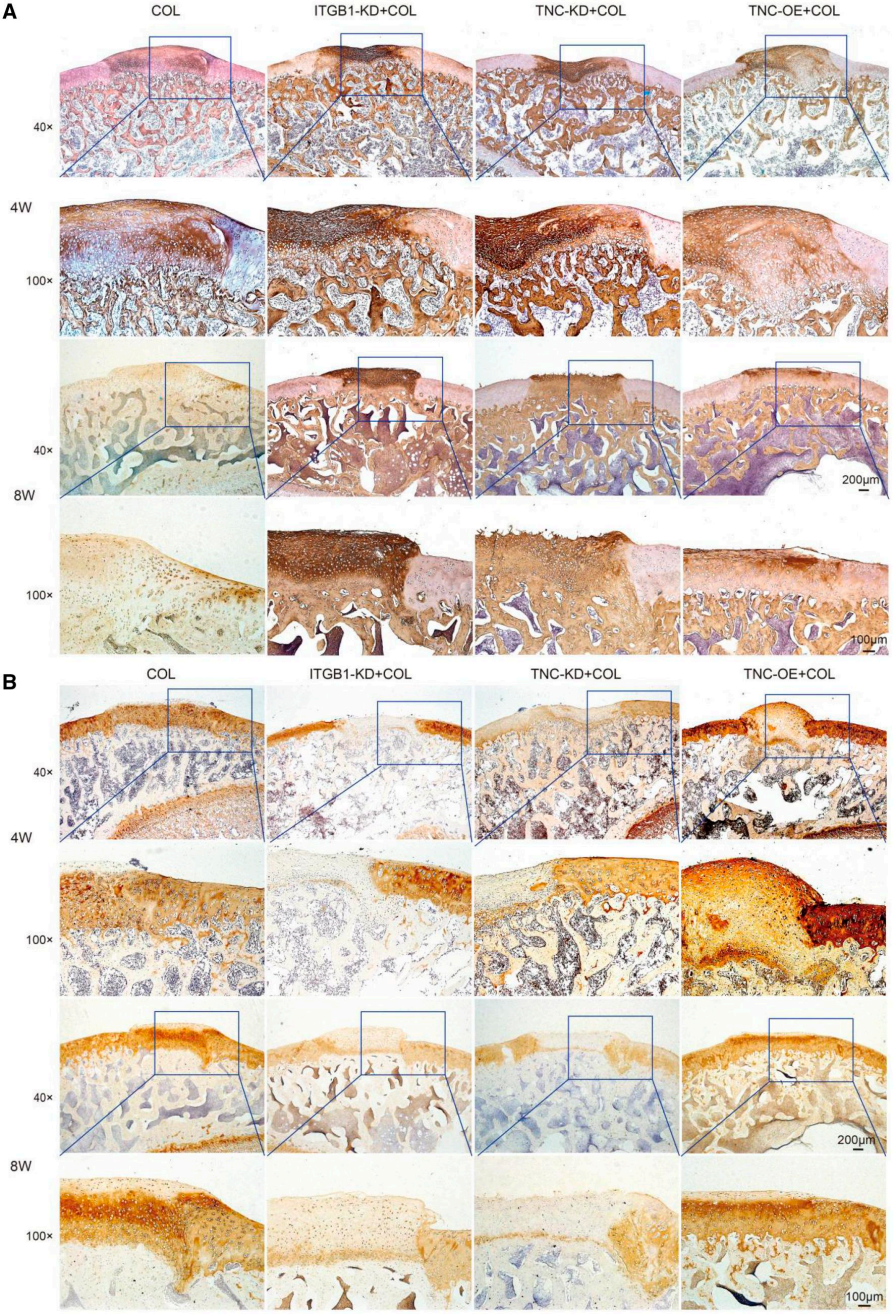
Immunohistochemical staining for COL1A1 (**A**) and COL2A1 (**B**) in repaired cartilage after 4 and 8 weeks post-surgery (scale bars: 200 and100 μm).

**Figure 9.**
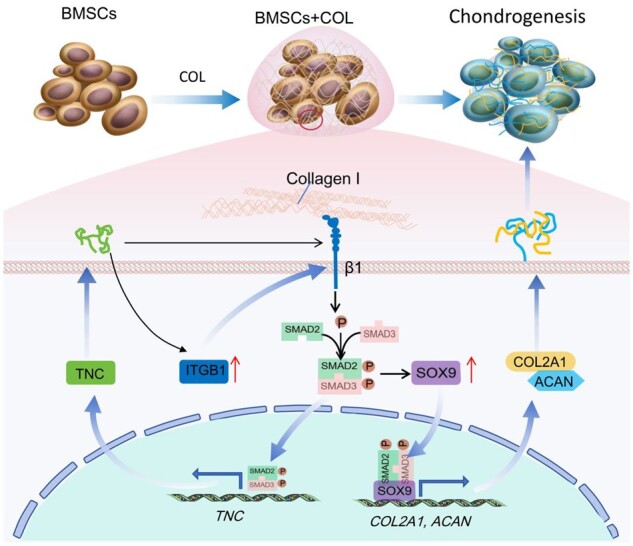
Collagen hydrogel acts on ITGB1, followed by activating the ITGB1, then induces phosphorylation of SMAD2/3, the p-SMAD2/3 enters into the nucleus and binds to the promoter regions of TNC to initiate its transcription [58]. TNC increases the expression of ITGB1 and further induces activation of ITGB1 and SMAD2/3. p-SMAD2/3 promotes the stability and accumulation of SOX9 protein and forms a transcriptional complex with SOX9, promoting the expression of cartilage-related genes, like COL2A1, etc.

The expression of fibrocartilage-related marker (COL1A1) and hyaline cartilage-related marker (COL2A1) in the regenerated tissues was detected by immunohistochemical staining. In the induction of collagen hydrogel, COL1A1 was negatively stained and the COL2A1 was positively stained after 4 and 8 weeks of treatment (Figure 8A and B). The opposite tendency of COL1A1 and COL2A1 expression was observed by knockdown of ITGB1 or TNC. Overexpression of TNC recovered the expression tendency of COL1A1 and COL2A1 close to the COL group. Studies have shown that ITGB1 was negatively correlated with the expression of COL1A1 [63], and the interaction between integrin β1 and collagen I could inhibit fibroblast proliferation [68]. ITGB1 and TNC may play a key role in hyaline-like cartilage regeneration induced by collagen hydrogel. The schematic diagram is shown in Figure 9.

## Conclusion

Chondrogenic differentiation of BMSCs induced by collagen hydrogel ITGB1 is mainly dependent on the ITGB1-mediated activation of TGF signaling. The phosphorylation of SMAD2/3 subsequently initiated the transcription of TNC, which in turn promoted ITGB1-mediated activation of TGF-SMAD2/3 signaling to extend the chondrogenic induction effect of collagen hydrogel. These may provide guidance to the design of ECM-mimicked chondrogenic materials for cartilage defect repair.

## Supplementary Material

rbae017_Supplementary_Data

## Data Availability

The full data of the research are contained in this article and the supplementary material.
