# Supplementary material for: Role of integrin β1 and tenascin C mediate TGF-SMAD2/3 signaling in chondrogenic differentiation of BMSCs induced by type I collagen hydrogel
Source: Regen Biomater. 2024 Feb 24;11:rbae017. doi: 10.1093/rb/rbae017 (PMC10960929; doi:10.1093/rb/rbae017)
Supplement: rbae017_Supplementary_Data [file rbae017_supplementary_data.docx]

**Role of Integrin β1 and** **Tenascin C Mediate TGF-SMAD2/3 Signaling in Chondrogenic Differentiation of BMSCs Induced by Type I Collagen Hydrogel**

**Contributor Information**

Yuanjun Huang, Guangxi Engineering Center in Biomedical Materials for Tissue and Organ Regeneration, The First Affiliated Hospital of Guangxi Medical University. Collaborative Innovation Centre of Regenerative Medicine and Medical BioResource Development and Application Co-Constructed by the Province and Ministry, Guangxi Medical University, No. 22 Shuangyong Road, Qingxiu District, Nanning City, 530021, China. Department of Trauma Orthopedic and Hand Surgery, The First Affiliated Hospital of Guangxi Medical University. No. 6 Shuangyong Road, Qingxiu District, Nanning City, 530021, China.

Miao Sun, Guangxi Engineering Center in Biomedical Materials for Tissue and Organ Regeneration, The First Affiliated Hospital of Guangxi Medical University. Collaborative Innovation Centre of Regenerative Medicine and Medical BioResource Development and Application Co-Constructed by the Province and Ministry, Guangxi Medical University, No. 22 Shuangyong Road, Qingxiu District, Nanning City, 530021, China.

Zhenhui Lu, Guangxi Engineering Center in Biomedical Materials for Tissue and Organ Regeneration, The First Affiliated Hospital of Guangxi Medical University. Collaborative Innovation Centre of Regenerative Medicine and Medical BioResource Development and Application Co-Constructed by the Province and Ministry, Guangxi Medical University. Guangxi Key Laboratory of Regenerative Medicine, The First Affiliated Hospital of Guangxi Medical University. Life Science Institute, Guangxi Medical University, No. 22 Shuangyong Road, Qingxiu District, Nanning City, 530021, China.

Qiuling Zhong, Guangxi Engineering Center in Biomedical Materials for Tissue and Organ Regeneration, The First Affiliated Hospital of Guangxi Medical University. Collaborative Innovation Centre of Regenerative Medicine and Medical BioResource Development and Application Co-Constructed by the Province and Ministry, Guangxi Medical University. No. 22 Shuangyong Road, Qingxiu District, Nanning City, 530021, China.

Manli Tan, Guangxi Engineering Center in Biomedical Materials for Tissue and Organ Regeneration, The First Affiliated Hospital of Guangxi Medical University. Collaborative Innovation Centre of Regenerative Medicine and Medical BioResource Development and Application Co-Constructed by the Province and Ministry, Guangxi Medical University. Guangxi Key Laboratory of Regenerative Medicine, The First Affiliated Hospital of Guangxi Medical University. Life Science Institute, Guangxi Medical University, No. 22 Shuangyong Road, Qingxiu District, Nanning City, 530021, China.

Qingjun Wei, Department of Trauma Orthopedic and Hand Surgery, The First Affiliated Hospital of Guangxi Medical University. No. 6 Shuangyong Road, Qingxiu District, Nanning City, 530021, China.

Li Zheng, Guangxi Engineering Center in Biomedical Materials for Tissue and Organ Regeneration, The First Affiliated Hospital of Guangxi Medical University. Collaborative Innovation Centre of Regenerative Medicine and Medical BioResource Development and Application Co-Constructed by the Province and Ministry, Guangxi Medical University. Guangxi Key Laboratory of Regenerative Medicine, The First Affiliated Hospital of Guangxi Medical University. Life Science Institute, Guangxi Medical University, No. 22 Shuangyong Road, Qingxiu District, Nanning City, 530021, China.

Super-enhancers

| #SUPER_ENHANCER_ID: unique ID for each super enhancer region | | | | | | | | | | | | | | | | |  |
| --- | --- | --- | --- | --- | --- | --- | --- | --- | --- | --- | --- | --- | --- | --- | --- | --- | --- |
| #SUPER_ENHANCER_CHRM: the chromosome which a super enhancer region lies in | | | | | | | | | | | | | | | | |  |
| #SUPER_ENHANCER_START: start site of a super enhancer region | | | | | | | | | | | | | | | | |  |
| #SUPER_ENHANCER_STOP: stop site of a super enhancer region | | | | | | | | | | | | | | | | |  |
| #CONSTITUENT_NUM: the number of constituent regions in a super enhancer region | | | | | | | | | | | | | | | | |  |
| #CONSTITUENT_SIZE: the length of an super enhancer | | | | | | | | | | | | | | | | |  |
| #Col-7days-IP: the signals of IP sample | | | | | | | | | | | | | | | | |  |
| #Col-7days-input: the signals of Input sample | | | | | | | | | | | | | | | | |  |
| #Col-7days-IP - Col-7days-input: the signals of IP sample subtracted by Input sample | | | | | | | | | | | | | | | | |  |
| #ClOEST: Closest gene is the gene which is the nearest to a super-enhancer. | | | | | | | | | | | | | | | | |  |
| #GENE_NAME: Name of a gene. | | | | | | | | | | | | | | | | |  |
| #REFSEQID: Accession number of a gene transcript. | | | | | | | | | | | | | | | | |  |
| #Chrom: the chromosome which a gene transcript lies in. | | | | | | | | | | | | | | | | |  |
| #Strand: Strand of the transcript. | | | | | | | | | | | | | | | | |  |
| #txStart(transcript): The start site of the transcript. | | | | | | | | | | | | | | | | |  |
| #txEnd(transcript): The termination site of the transcript. | | | | | | | | | | | | | | | | |  |
| #enhancerRank: The rank of the super-enhancer signals. | | | | | | | | | | | | | | | | |  |
| #isSuper: Represent whether it is a super-enhancer. | | | | | | | | | | | | | | | | |  |
| #contained_peaks: The ChIP-seq peaks region contained in a super-enhancer. | | | | | | | | | | | | | | | | |  |
|  |  |  |  |  |  |  |  |  |  |  |  |  |  |  |  |  |  |
|  |  |  |  |  |  |  |  |  |  |  |  |  |  |  |  |  |  |
| **ENHANCER_ID** | **ENHANCER_CHRM** | **ENHANCER_START** | **ENHANCER_STOP** | **CONSTITUENT_NUM** | **CONSTITUENT_SIZE** | **Col-7days-IP** | **Col-7days-input** | **Col-7days-IP - Col-7days-input** | **CLOSEST_GENE** | **REFSEQID** | **Chrom** | **Strand** | **txStart (transcript)** | **txEnd (transcript)** | **enhancerRank** | **is Super** | **contained_peaks** |
| 1_MACS_peak_5241_lociStitched | chr9 | 88977048 | 88978475 | 1 | 1428 | 40011.0822 | 12686.8862 | 27324.196 | SMAD2 | XM_002713521.3 | chr9 | - | 89082150 | 89013099 | 4 | 1 | chr9:88977048-88978475 |
| 2_MACS_peak_835_lociStitched | chr12 | 1523266 | 1535851 | 2 | 2750 | 12381.123 | 1671.288 | 10709.835 | BMP6 | XM_008262354.2 | chr12 | + | 1470750 | 1647032 | 8 | 1 | chr12:1523266-1525040, chr12:1534877-1535851 |
| 2_MACS_peak_1838_lociStitched | chr14 | 91432864 | 91437271 | 2 | 2215 | 2314.5564 | 485.2107 | 1829.3457 | DLG1 | XM_017347003.1 | chr14 | + | 91533795 | 91782008 | 42 | 1 | chr14:91432864-91434350, chr14:91436544-91437271 |
| 2_MACS_peak_2584_lociStitched | chr17 | 52752041 | 52765479 | 2 | 2111 | 203482.2274 | 63221.7586 | 140260.4688 | NPAS3 | XM_017348254.1 | chr17 | + | 52790420 | 53594744 | 1 | 1 | chr17:52752041-52752875, chr17:52764204-52765479 |
| 1_MACS_peak_200_lociStitched | chr1 | 75304611 | 75306335 | 1 | 1725 | 56067.066 | 13504.092 | 42562.974 | HABP4 | XM_017347490.1 | chr1 | + | 75279954 | 75322772 | 2 | 1 | chr1:75304611-75306335 |
| 1_MACS_peak_2705_lociStitched | chr18 | 17162883 | 17164710 | 1 | 1828 | 39194.0829 | 10789.7139 | 28404.369 | C18H10orf35 | XM_002718441.3 | chr18 | - | 17220260 | 17218212 | 3 | 1 | chr18:17162883-17164710 |
| 2_MACS_peak_4356_lociStitched | chr6 | 13389617 | 13402614 | 2 | 2000 | 22309.3505 | 7848.8883 | 14460.4622 | OTOA | XM_017342524.1 | chr6 | + | 13338633 | 13408487 | 5 | 1 | chr6:13389617-13390596, chr6:13401595-13402614 |
| 1_MACS_peak_619_lociStitched | chr10 | 45511365 | 45512565 | 1 | 1201 | 16553.76 | 4483.92 | 12069.84 | VOPP1 | XM_008261854.2 | chr10 | + | 45520132 | 45562216 | 6 | 1 | chr10:45511365-45512565 |
| 1_MACS_peak_2277_lociStitched | chr16 | 11098222 | 11099764 | 1 | 1543 | 16446.5094 | 4633.5558 | 11812.9536 | PRKCQ | XM_017347655.1 | chr16 | - | 10879141 | 10726383 | 7 | 1 | chr16:11098222-11099764 |
| 1_MACS_peak_1605_lociStitched | chr13 | 142601520 | 142602285 | 1 | 766 | 12934.926 | 3379.3875 | 9555.5385 | LOC103350348 | XM_017345595.1 | chr13 | - | 142878674 | 142460633 | 9 | 1 | chr13:142601520-142602285 |
| 1_MACS_peak_268_lociStitched | chr1 | 96522754 | 96524176 | 1 | 1423 | 9409.5162 | 82.476 | 9327.0402 | SORL1 | XM_008258525.2 | chr1 | - | 96539436 | 96383447 | 10 | 1 | chr1:96522754-96524176 |
| 1_MACS_peak_2252_lociStitched | chr16 | 242385 | 244133 | 1 | 1749 | 11546.0644 | 2956.5672 | 8589.4972 | OTUD1 | XM_017347598.1 | chr16 | + | 79825 | 81368 | 11 | 1 | chr16:242385-244133 |
| 1_MACS_peak_1207_lociStitched | chr12 | 149534878 | 149536927 | 1 | 2050 | 10082.3094 | 2946.0522 | 7136.2572 | LOC100357076 | XM_008263743.2 | chr12 | - | 149541325 | 149536517 | 12 | 1 | chr12:149534878-149536927 |
| 3_MACS_peak_3415_lociStitched | chr2 | 155391904 | 155417600 | 3 | 9104 | 14489.9744 | 8723.792 | 5766.1824 | LOC100344894 | XM_017340836.1 | chr2 | + | 155325582 | 155369600 | 13 | 1 | chr2:155391904-155393847, chr2:155401650-155405236, chr2:155414028-155417600 |
| 1_MACS_peak_4430_lociStitched | chr7 | 14172642 | 14173918 | 1 | 1277 | 5821.8776 | 151.9716 | 5669.906 | MEST | XM_002712095.3 | chr7 | - | 14148259 | 14134936 | 14 | 1 | chr7:14172642-14173918 |
| 1_MACS_peak_4601_lociStitched | chr7 | 100424807 | 100425948 | 1 | 1142 | 19358.6624 | 13965.2695 | 5393.3929 | PSMD14 | XM_017342893.1 | chr7 | + | 100434306 | 100546114 | 15 | 1 | chr7:100424807-100425948 |
| 1_MACS_peak_1014_lociStitched | chr12 | 69917873 | 69918676 | 1 | 804 | 16484.2249 | 11097.3797 | 5386.8452 | UBE3D | XM_008263151.2 | chr12 | - | 70021504 | 69852462 | 16 | 1 | chr12:69917873-69918676 |
| 1_MACS_peak_3480_lociStitched | chr20 | 13702594 | 13703941 | 1 | 1348 | 4717.0593 | 795.5382 | 3921.5211 | SLC24A4 | XM_017349475.1 | chr20 | - | 13638014 | 13492256 | 17 | 1 | chr20:13702594-13703941 |
| 2_MACS_peak_4213_lociStitched | chr5 | 379747 | 398236 | 2 | 7563 | 11474.2734 | 7584.1878 | 3890.0856 | LEUTX | XM_017342186.1 | chr5 | - | 379949 | 378090 | 18 | 1 | chr5:379747-385138, chr5:396066-398236 |
| 1_MACS_peak_789_lociStitched | chr11 | 77341337 | 77343100 | 1 | 1764 | 3884.9468 | 341.3168 | 3543.63 | SMIM15 | XM_017344737.1 | chr11 | - | 77198136 | 77194614 | 19 | 1 | chr11:77341337-77343100 |
| 3_MACS_peak_1604_lociStitched | chr13 | 142424692 | 142430755 | 3 | 2216 | 5309.3691 | 1917.1206 | 3392.2485 | CAMTA1 | XM_008265979.2 | chr13 | - | 142157727 | 142064538 | 20 | 1 | chr13:142424692-142425679, chr13:142429251-142429815, chr13:142430093-142430755 |
| 1_MACS_peak_3601_lociStitched | chr3 | 4862645 | 4863849 | 1 | 1205 | 3364.2168 | 34.1936 | 3330.0232 | PRR16 | XM_002710144.3 | chr3 | + | 4906205 | 5093709 | 21 | 1 | chr3:4862645-4863849 |
| 1_MACS_peak_3216_lociStitched | chr2 | 79126540 | 79127555 | 1 | 1016 | 4499.089 | 1214.143 | 3284.946 | LOC100344388 | XM_008252530.2 | chr2 | - | 79170829 | 79168795 | 22 | 1 | chr2:79126540-79127555 |
| 2_MACS_peak_4370_lociStitched | chr6 | 18067524 | 18084184 | 2 | 4925 | 6823.936 | 3576.902 | 3247.034 | GSG1L | XM_008257925.2 | chr6 | - | 18074452 | 17890153 | 23 | 1 | chr6:18067524-18071055, chr6:18082792-18084184 |
| 1_MACS_peak_2668_lociStitched | chr18 | 6143105 | 6149486 | 1 | 6382 | 10025.8272 | 7216.2729 | 2809.5543 | LOC108175357 | XM_008269841.1 | chr18 | + | 6081223 | 6092104 | 24 | 1 | chr18:6143105-6149486 |
| 1_MACS_peak_1258_lociStitched | chr13 | 8330656 | 8331507 | 1 | 852 | 2734.4332 | 103.5667 | 2630.8665 | WDR60 | XM_002715015.3 | chr13 | - | 8441572 | 8358239 | 25 | 1 | chr13:8330656-8331507 |
| 3_MACS_peak_930_lociStitched | chr12 | 31097496 | 31108569 | 3 | 3815 | 4182.2721 | 1563.5076 | 2618.7645 | LOC100349504 | XM_002714672.3 | chr12 | - | 30999674 | 30991512 | 26 | 1 | chr12:31097496-31098495, chr12:31099674-31101564, chr12:31107646-31108569 |
| 1_MACS_peak_472_lociStitched | chr1 | 173815411 | 173816343 | 1 | 933 | 3271.32 | 708.8792 | 2562.4408 | ELF5 | XM_017345905.1 | chr1 | - | 173811260 | 173767766 | 27 | 1 | chr1:173815411-173816343 |
| 2_MACS_peak_2741_lociStitched | chr18 | 36410114 | 36425538 | 2 | 4125 | 5153.1584 | 2629.792 | 2523.3664 | RNLS | XM_008270155.2 | chr18 | - | 36495382 | 36199049 | 28 | 1 | chr18:36410114-36413002, chr18:36424303-36425538 |
| 2_MACS_peak_1280_lociStitched | chr13 | 16574596 | 16589687 | 2 | 3448 | 9632.5853 | 7261.7892 | 2370.7961 | PAXIP1 | XM_008263932.1 | chr13 | - | 16509855 | 16443248 | 29 | 1 | chr13:16574596-16577278, chr13:16588923-16589687 |
| 1_MACS_peak_1424_lociStitched | chr13 | 71087588 | 71088372 | 1 | 785 | 10711.008 | 8434.8208 | 2276.1872 | FNBP1L | XM_017346075.1 | chr13 | - | 71064510 | 70940122 | 30 | 1 | chr13:71087588-71088372 |
| 1_MACS_peak_2648_lociStitched | chr17 | 82820629 | 82825852 | 1 | 5224 | 5428.2639 | 3224.6802 | 2203.5837 | LOC103351283 | XM_017348352.1 | chr17 | - | 82944933 | 82943580 | 31 | 1 | chr17:82820629-82825852 |
| 1_MACS_peak_2662_lociStitched | chr18 | 4319733 | 4322074 | 1 | 2342 | 7433.3773 | 5245.2446 | 2188.1327 | LOC100355721 | XM_017348673.1 | chr18 | - | 4414070 | 4413287 | 32 | 1 | chr18:4319733-4322074 |
| 2_MACS_peak_2938_lociStitched | chr19 | 29578747 | 29583401 | 2 | 4637 | 3824.6572 | 1669.8552 | 2154.802 | RAD51C | XM_008271207.2 | chr19 | - | 29581120 | 29540718 | 33 | 1 | chr19:29578747-29580642, chr19:29580661-29583401 |
| 1_MACS_peak_5082_lociStitched | chr9 | 18370379 | 18371781 | 1 | 1403 | 2834.2832 | 701.9814 | 2132.3018 | LOC100342543 | XM_008260706.2 | chr9 | + | 18347941 | 18349048 | 34 | 1 | chr9:18370379-18371781 |
| 1_MACS_peak_3015_lociStitched | chr19 | 50801015 | 50802558 | 1 | 1544 | 2343.1998 | 221.8834 | 2121.3164 | CACNG5 | XM_017349287.1 | chr19 | - | 50568542 | 50558717 | 35 | 1 | chr19:50801015-50802558 |
| 2_MACS_peak_283_lociStitched | chr1 | 99025887 | 99038689 | 2 | 1900 | 3448.8588 | 1330.1278 | 2118.731 | CEP164 | XM_017343150.1 | chr1 | - | 99119399 | 99043896 | 36 | 1 | chr1:99025887-99026841, chr1:99037745-99038689 |
| 2_MACS_peak_2916_lociStitched | chr19 | 22543198 | 22556666 | 2 | 2827 | 3569.02 | 1461.278 | 2107.742 | SPACA3 | XM_017349341.1 | chr19 | + | 22496177 | 22505062 | 37 | 1 | chr19:22543198-22544455, chr19:22555098-22556666 |
| 1_MACS_peak_1307_lociStitched | chr13 | 31533294 | 31536358 | 1 | 3065 | 2993.528 | 1054.016 | 1939.512 | LOC100338913 | XM_002715249.3 | chr13 | - | 31550198 | 31543346 | 38 | 1 | chr13:31533294-31536358 |
| 1_MACS_peak_486_lociStitched | chr1 | 182731663 | 182733268 | 1 | 1606 | 2189.3805 | 257.442 | 1931.9385 | LOC103351772 | XM_008272387.1 | chr1 | - | 182737036 | 182730974 | 39 | 1 | chr1:182731663-182733268 |
| 1_MACS_peak_2272_lociStitched | chr16 | 6396871 | 6402135 | 1 | 5265 | 3665.3232 | 1774.4944 | 1890.8288 | LOC103350879 | XM_008268033.2 | chr16 | + | 6350946 | 6351450 | 40 | 1 | chr16:6396871-6402135 |
| 2_MACS_peak_4906_lociStitched | chr8 | 53759647 | 53770632 | 2 | 1701 | 2964.8515 | 1102.894 | 1861.9575 | NAA16 | XM_017343501.1 | chr8 | - | 53758048 | 53678007 | 41 | 1 | chr8:53759647-53760404, chr8:53769690-53770632 |
|  |  |  |  |  |  |  |  |  |  |  |  |  |  |  |  |  |  |
